# *Staphylococcus saccharolyticus* Isolated From Blood Cultures and Prosthetic Joint Infections Exhibits Excessive Genome Decay

**DOI:** 10.3389/fmicb.2019.00478

**Published:** 2019-03-12

**Authors:** Holger Brüggemann, Anja Poehlein, Elzbieta Brzuszkiewicz, Carsten Scavenius, Jan J. Enghild, Munir A. Al-Zeer, Volker Brinkmann, Anders Jensen, Bo Söderquist

**Affiliations:** ^1^Department of Biomedicine, Aarhus University, Aarhus, Denmark; ^2^Department of Genomic and Applied Microbiology, Institute of Microbiology and Genetics, University of Göttingen, Göttingen, Germany; ^3^Department of Molecular Biology and Genetics, Aarhus University, Aarhus, Denmark; ^4^Department of Applied Biochemistry, Institute of Biotechnology, Technical University of Berlin, Berlin, Germany; ^5^Microscopy Core Facility, Max Planck Institute for Infection Biology, Berlin, Germany; ^6^Department of Laboratory Medicine, Clinical Microbiology, Faculty of Medicine and Health, Örebro University, Örebro, Sweden

**Keywords:** *Staphylococcus*, *Staphylococcus saccharolyticus*, coagulase-negative staphylococci, prosthetic joint infection, slow-growing bacteria, genome, genome decay, hyaluronic acid lyase

## Abstract

The slow-growing, anaerobic, coagulase-negative species *Staphylococcus saccharolyticus* is found on human skin and in clinical specimens but its pathogenic potential is unclear. Here, we investigated clinical isolates and sequenced the genomes of seven strains of *S. saccharolyticus*. Phylogenomic analyses showed that the closest relative of *S. saccharolyticus* is *Staphylococcus capitis* with an average nucleotide identity of 80%. Previously sequenced strains assigned to *S. saccharolyticus* are misclassified and belong to *S. capitis*. Based on single nucleotide polymorphisms of the core genome, the population of *S. saccharolyticus* can be divided into two clades that also differ in a few larger genomic islands as part of the flexible genome. An unexpected feature of *S. saccharolyticus* is extensive genome decay, with over 300 pseudogenes, indicating ongoing reductive evolution. Many genes of the core metabolism are not functional, rendering the species auxotrophic for several amino acids, which could explain its slow growth and need for fastidious growth conditions. Secreted proteins of *S. saccharolyticus* were determined; they include stress response proteins such as heat and oxidative stress-related factors, as well as immunodominant staphylococcal surface antigens and enzymes that can degrade host tissue components. The strains secrete lipases and a hyaluronic acid lyase. Hyaluronidase as well as urease activities were detected in biochemical assays, with clade-specific differences. Our study revealed that *S. saccharolyticus* has adapted its genome, possibly due to a recent change of habitat; moreover, the data imply that the species has tissue-invasive potential and might cause prosthetic joint infections.

## Introduction

Coagulase-negative staphylococci (CoNS) are a very heterogeneous group of bacteria; several CoNS colonize the human skin and are part of the normal microbiota. The coagulase reaction distinguishes them from the clinically important species *Staphylococcus aureus* that possesses an arsenal of virulence factors, in contrast to most CoNS that are less frequently associated with infections in humans. As such, CoNS are often considered as non-pathogenic bacteria. However, in the last decades it has become more evident that some CoNS are important (nosocomial) opportunistic pathogens, such as *Staphylococcus epidermidis*, *Staphylococcus haemolyticus*, and *Staphylococcus capitis*, as causative agents of foreign body infections, e.g., orthopedic implant-associated infections, as well as infections in immunocompromised patients and neonates, and *Staphylococcus saprophyticus* as a causative agent of lower urinary tract infections (Huebner and Goldmann, 1999; Becker et al., 2014; Butin et al., 2016; Arciola et al., 2018). There is a large variation among the different CoNS species regarding their virulence potential with substantial differences also on strain level (Otto, 2004; Becker et al., 2014; Cameron et al., 2015).

Almost all CoNS are facultative anaerobes; only very few strains identified so far have been classified as anaerobic, including one strain of *S. epidermidis* and strains of the species *Staphylococcus saccharolyticus*, formerly called *Peptococcus saccharolyticus* (Evans et al., 1978; Rowlinson et al., 2006). This species was found to be part of the microbiota of the skin and detected by prolonged (4–7 days) anaerobic cultivation in rich media. It apparently rarely causes infections in humans. Consequently, in the past, findings of *S. saccharolyticus* in blood cultures were considered as contaminants (Hitzenbichler et al., 2017). However, the organism was detected in blood samples of 16 inpatients in an apparent hospital outbreak of bacteremia in Germany (Steinbrueckner et al., 2001). Moreover, *S. saccharolyticus* has been reported in case studies as an etiologic agent of infective endocarditis and bone and joint infections such as shoulder synovitis and vertebral osteomyelitis (Westblom et al., 1990; Godreuil et al., 2005; Mikhael et al., 2009; Schneeberger et al., 2012). There are no reports of implant-associated infections, except one case of prosthetic valve endocarditis (Krishnan et al., 1996). In their systematic literature review on anaerobic prosthetic joint infections (PJIs) Shah et al. (2015) did not report any cases of PJI caused by *S. saccharolyticus*.

Taken together, knowledge about this anaerobic CoNS species is fragmentary. We therefore investigated clinical *S. saccharolyticus* isolates that have been detected in blood cultures and PJI specimens. We sequenced and analyzed their genomes and performed biochemical tests. It was found that these isolates were actually not identical to previously sequenced *S. saccharolyticus* strains; the latter have been misclassified and were largely identical with *S. capitis*. Thus, we sequenced and analyzed the (pan-)genome of *S. saccharolyticus*. Our study describes interesting features of this species such as urease and hyaluronidase activities as well as its extensive genome decay and sheds light on a taxonomic inaccurateness among CoNS, including clinically relevant species.

## Materials and Methods

### Bacterial Strains

The study was approved by the Regional Ethical Review Board of Uppsala, Sweden (reference 2016/457/1, amendment 2018-02-21). Informed consent was obtained from each patient for collecting information to the Swedish national quality registers and no additional informed consent was required according to the approval by the Regional Ethical Review Board. We confirm that all research was performed in accordance with relevant guidelines and regulations.

Twenty strains have been used in this study (Supplementary Table S1A). These strains were isolated from blood cultures or PJIs of patients at the Örebro University Hospital, Sweden. All strains were grown under anaerobic conditions on FAA plates (4.6% LAB 90 Fastidious Anaerobe Agar, LAB M, Heywood, United Kingdom) supplemented with 5% horse blood (v/v) and incubated at 37°C in anaerobic conditions for 7 days. All 20 strains were identified as *S. saccharolyticus*, using MALDI-TOF MS (Microflex LT and Biotyper 3.1, Bruker Daltonics).

### DNA Extraction and Genome Sequencing

Genomic DNA was isolated using the MasterPure Gram-positive DNA Purification Kit (EpiCentre MGP04100) according to the manufacturer’s instructions. The purity and quality of the gDNA were assessed on a 1% agarose gel and with a nanodrop apparatus (Thermo Fisher Scientific). The extracted DNA was used to generate Illumina shotgun paired-end sequencing libraries using the Nextera^©^ XT Dn NA Sample PreparatioKit and the Nextera^©^ XT Index Kit as recommended by the manufacturer. For strains 05B0362, 12B0021 and DVP3-16-6167 the library preparation failed with the above-described kit. For these strains we used the Nextera^©^ DNA Sample Preparation Kit and Nextera Index Kit, which is recommended to be used for genomes larger than 8 Mb or metagenomes but not for genomes with expected genomes sizes of around 2.5 Mb. The libraries have been sequenced on a MiSeq instrument and the MiSeq reagent kit version 3 as recommended by the manufacturer (Illumina, San Diego, CA, United States). For quality-filtering of the raw reads, Trimmomatic version 0.36 was used and the assemblies were performed with the SPAdes genome assembler software (version 3.11.1) (Bankevich et al., 2012; Bolger et al., 2014). QualiMap v.2.2.1 was used for validation of the assemblies (Okonechnikov et al., 2016). The assemblies resulted in a coverage of the genomes between 167- and 289-fold. Accordingly, the contig numbers were low, between 10 and 13. Further information is listed in Table 1 and Supplementary Table S1A.

**Table 1:** Features of draft genomes of *Staphylococcus saccharolyticus*.

Strain	Genome size (kb)	G+C (%)	Contigs	N50 (kb)	CDS (coding density in %)	corrected CDS^∗^ (coding density in %)	putative pseudo-genes
05B0362	2,349	32.0	10	768	2,737 (82.1)	2,280 (74.2)	307
12B0021	2,349	32.0	11	768	2,741 (82.0)	2,277 (74.1)	309
13T0028	2,332	32.0	13	372	2,715 (82.2)	2,253 (74.2)	306
DVP3-16-6167	2,350	32.0	13	768	2,741 (82.1)	2,283 (74.2)	306
DVP2-17-2406	2,373	32.0	11	1,222	2,678 (82.1)	2,257 (74.3)	291
DVP4-17-2404	2,373	32.0	11	1,222	2,677 (82.1)	2,256 (74.3)	289
DVP5-16-4677	2,376	32.0	12	1,223	2,691 (82.2)	2,256 (74.2)	299

ATCC14953_ DRR015951	2,351	32.0	61	73	2,736 (81.3)	2,279 (73.6)	307
ATCC14953_ SRR5029787	2,348	32.0	57	77	2,734 (81.3)	2,279 (73.6)	304


*^∗^Automatically predicted CDS (RAST annotation) minus the number of pseudo-ORFs.*

### Genome Comparison, Phylogenomic and Other Bioinformatic Analyses

For comparative analyses, we used genomes of other previously sequenced CoNS strains (Supplementary Table S1B); these included the genomes of strains that have previously been (incorrectly) assigned to *S. saccharolyticus* (strain KR: GenBank accession number NDFI00000000; strain OG2-1: NDFK00000000, strain OG2-2: NDFL00000000). In addition, we used the following high-quality genomes of *S. capitis* strains: AYP1020 (GenBank accession number: CP007601), TW2795 (AP014956), FDAARGOS_378 (CP023966), CR01 (CBUB000000000), C87 (ACRH00000000). Regarding *S. epidermidis* we used the following genomes: ATCC12228 (NC_004461.1), RP62A (NC_002976.3), SEI (NZ_CP009046.1), 14.1.R1 (NZ_CP018842.1), AU23 (LNUS00000000.1), FS1 (LOAT00000000.1).

Raw sequence data of the *S. saccharolyticus* type strain ATCC 14953 (=DSM 20359 = JCM 1768 = NCTC11807) was retrieved from the Sequence Read Archive (SRA) [two projects; (1) accession: SRX2355498, run: SRR5029787, 524.4M bases; (2) accession: DRX014323, run: DRR015951, 693M bases] and assembled with SPAdes genome assembler software (version 3.11.1) (Bankevich et al., 2012).

For phylogenomic analyses, the core genome was identified and aligned with Parsnp, a program that is part of the Harvest software package (Treangen et al., 2014). Parsnp aligns microbial genomes based on a suffix graph data structure; the output is a core-genome alignment that contains all single nucleotide polymorphisms (SNPs), Indels, and structural variation within the core genome. Parsnp is further quality-filtering SNPs; only reliable core-genome SNPs are considered for reconstruction of the whole-genome phylogeny that can be visualized with Gingr, another program of the Harvest software package. As a second phylogenomic program we used CSI phylogeny (Kaas et al., 2014). To calculate the average nucleotide identity (ANI) between genomes the program JSpecies was used (Richter and Rosselló-Móra, 2009). Jspecies calculates the ANI between the genomes in a pairwise comparison using BLAST.

Gene prediction and annotation of all genomes were done with RAST (Aziz et al., 2008). Phylogenetic trees were visualized using Mega v7 (Tamura et al., 2013) and Interactive Tree Of Life (iTOL^1^). For comparative genome analyses and visualization, the program BRIG was used (Alikhan et al., 2011). To determine orthologous genes among the CoNS strains we used the tool Proteinortho (Lechner et al., 2011). As promoter prediction tool we used BPROM (Solovyev and Salamov, 2011).

### Biochemical Tests

Two test systems, API^®^ 20A (BioMérieux) and RapID^TM^ ANA II (Remel/Thermo Fisher), were used according to the instructions of the manufacturers. In brief, *S. saccharolyticus* strains were grown on FAA agar plates under anaerobic conditions for 7 days; cells were harvested and resuspended in the test system’s recommended inoculation fluids in the desired densities. For the RapID^TM^ ANA II kit the bacterial suspension had a visual turbidity equal to a no. 4 McFarland turbidity standard. After inoculation, the RapID^TM^ ANA II panel was incubated at 37°C for 5 h. The inoculated API 20A kit panel was incubated for 48 h. Additional substances were added after inoculation, and results were interpreted as described in the instructions of the manufacturers.

### Hyaluronidase Plate Assay

Hyaluronic acid (HA)-containing plates were prepared according to the method of Smith and Willett with some modifications (Smith and Willett, 1968). BHI medium was mixed with 2% (wt/vol) of Noble agar (Difco, Thermo-Fisher Scientific, Waltham, MA, United States) and autoclaved. Two hundred milliliters of a 0.2% stock solution of HA sodium salt (Carbosynth, Compton, United Kingdom) was added to the cooled media together with 200 mL of a 5% (wt/vol) stock solution of bovine serum albumin (BSA) under constant stirring. The media was poured on plates and stored at 4°C. Both HA and BSA were dissolved in water, sterile-filtered, and added to the agar medium. Colonies of *S. saccharolyticus* (harvested from FAA plates after 7 days of anaerobic incubation) were point-inoculated onto the surface of HA plates.

Plates were incubated for 48 h under anaerobic condition at 37°C and subsequently flushed with 2N acetic acid for at least 30 min. Clear zones around colonies indicate HA degradation, since degraded HA does not precipitate under acidic conditions. Recombinant hyaluronidase from *Streptococcus pyogenes* (Sigma-Aldrich, St. Louis, MO, United States) was used as a positive control for HA degradation.

### Scanning Electron Microscopy

*Staphylococcus saccharolyticus* strains were incubated on FAA agar plates under anaerobic conditions. Cells were harvested at two time points (4 and 7 days of growth) and resuspended in 1 mL PBS, and washed twice in PBS with gentle centrifugation (1000 rpm, 5 min). Bacterial cells were then fixed with 2.5% glutaraldehyde, post-fixed using repeated incubations with 1% osmium tetroxide/1% tannic acid, dehydrated with a graded ethanol series, critical point dried and coated with 3 nm platinum/carbon. Specimens were analyzed in a Leo 1550 scanning electron microscope.

### Enrichment of Secreted Proteins of *S. saccharolyticus*

For the collection of extracellular, secreted proteins, *S. saccharolyticus* strains were grown in BHCY medium [BHI medium, supplemented with 0.5% (w/vol) yeast extract and 0.05% (w/vol) cysteine] for 4 and 7 days. The cultures were centrifuged for 30 min at 4,000 *g* and 4°C. Supernatant was filtered through a 0.22-μm-pore-size membrane filter to remove residual bacteria. Extracellular proteins were precipitated using a modified trichloroacetic acid (TCA) method (Komoriya et al., 1999). In brief, the supernatant filtrate was mixed with TCA to a final concentration of 10% and incubated overnight at 4°C on a tube rotator. The mixture was centrifuged for 20 min (20,000 *g* and 4°C) and the resulting pellet was resuspended in 1 ml of ice-cold acetone, transferred to Eppendorf tubes and submerged into an ultrasonic bath for 10 min. The resuspended pellet was washed twice with acetone and the resulting pellet was air-dried and stored at -80°C. The pellets were suspended in 8 M Urea 0.1 M ammonium bicarbonate, reduced in 5 mM DTT for 1 h and alkylated in 15 mM iodoacetamide for 1 h. The samples were then diluted 1:5 with 0.1 M ammonium bicarbonate and digested with trypsin at 37°C for 16 h. The tryptic peptides were isolated and desalted by micropurification using Empore^TM^ SPE Disks of C18 octadecyl packed in 10 μl pipette tips (Rappsilber et al., 2007).

### Mass Spectrometry

Proteins in the secreted fraction were identified using nano-electrospray ionization MS/MS (nanoESI-MS/MS) analyses, performed on an eksigent nanoLC 415 system (SCIEX) connected to a TripleTOF 6600 mass spectrometer (SCIEX). The trypsin-digested samples were suspended in 0.1% formic acid, injected, trapped and desalted on a precolumn. The peptides were eluted and separated on a 15 cm analytical column (75 μm i.d.), pulled in-house (P2000 laser puller, Sutter Instrument). Trap and analytical column were packed with ReproSil-Pur C18-AQ 3 μm resin (Dr. Maisch GmbH). Peptides were eluted from the analytical column at a flow rate of 250 nl/min using a 30 min gradient from 5 to 35% of solution B (0.1% formic acid, 100% acetonitrile). The collected MS files were converted to Mascot generic format (MGF) using the AB SCIEX MS Data Converter beta 1.1 (AB SCIEX) and the “protein pilot MGF” parameters. The generated peak lists were searched using an in-house Mascot search engine (Matrix Science) against a customized *S. saccharolyticus* protein fasta database. Search parameters were allowing one missed trypsin cleavage site and carbamidomethyl as a fixed modification with peptide tolerance and MS/MS tolerance set to 10 ppm and 0.1 Da, respectively.

## Results

### Genome Sequencing of Anaerobic Staphylococci Isolated From Prosthetic Joint Infections and Blood Cultures

Slow-growing (>7 days of primary cultivation) anaerobic bacterial isolates, obtained from blood culture samples and patient specimens at Örebro University Hospital, Sweden, are routinely identified by Matrix Assisted Laser Desorption Ionization Time-of-Flight mass spectrometry (MALDI-TOF MS). A retrospective search identified seven cases in which *S. saccharolyticus* was detected in blood cultures and eight cases of PJIs (three patients with shoulder PJI and five patients with hip PJI) where *S. saccharolyticus* was obtained from tissue biopsies, identified by MALDI-TOF MS to the species level. For some patients with PJIs more than one bacterial isolate was investigated, detected in multiple tissue biopsies. Altogether, 20 isolates, seven derived from blood cultures and 13 obtained from PJIs in the time-period 2013–2017 were investigated (Supplementary Table S1A).

Since knowledge about *S. saccharolyticus* is scarce, we decided to genome sequence these isolates to obtain insight into their properties and virulence potential. Genome sequencing of these 20 isolates turned out to be unexpectedly difficult by the standard DNA extraction and Illumina sequencing protocols. After changing the protocol (see section “Materials and Methods”) we obtained high-quality genomic data for seven out of 20 strains. Genome features are summarized in Table 1 and compared to closely related staphylococcal species (Supplementary Table S1B). All newly sequenced genomes had a G+C content of 32.0%, which is almost identical to the one of *S. epidermidis* (31.95%, on average of the genomes deposited in GenBank), but lower compared to *S. capitis* (32.9%) and previously sequenced genomes of three strains assigned to *S. saccharolyticus* (33.2%). The average genome size of the here sequenced strains was 2,357 kb (range: from 2,333 to 2,376 kb), thus substantially smaller than the ones of *S. capitis* (2,487 kb, in average of the genomes deposited in GenBank), *S. epidermidis* (2,548 kb) and also lower than the previously sequenced genomes of strains assigned to *S. saccharolyticus* (2,548 kb).

### Misclassification of Previously Sequenced Strains Assigned to *S. saccharolyticus*

We were surprised to see genomic differences between the here sequenced and the previously sequenced strains assigned to *S. saccharolyticu*s. The genomic differences suggested that the newly sequenced strains belong to a distinct phylogenetic clade. First, we compared the average nucleotide identities (ANIs) of the newly sequenced strains with previously sequenced staphylococci. The ANI within the seven sequenced strains was 98.8% (with a strain-specific ANI variation from 97.9 to 100%), indicating that these seven strains belong to the same species (Supplementary Table S2). However, the seven strains exhibited surprisingly low ANI, in average 79.5%, with their closest relatives, i.e., *S. capitis*, *S. epidermidis* and three strains assigned to *S. saccharolyticus.* The data further revealed that the latter three strains have been incorrectly assigned to *S. saccharolyticus*: the ANI of strains KR and OG1-2 with *S. capitis* was in average 97.8%, which indicates that these two strains belong to *S. capitis*. There is one exception: strain OG2-2 had an ANI of 83% with *S. capitis*; thus, it could represent a distinct species. However, it was not similar to the seven *S. saccharolyticus* genomes sequenced here (ANI below 80%).

**FIGURE 1:**
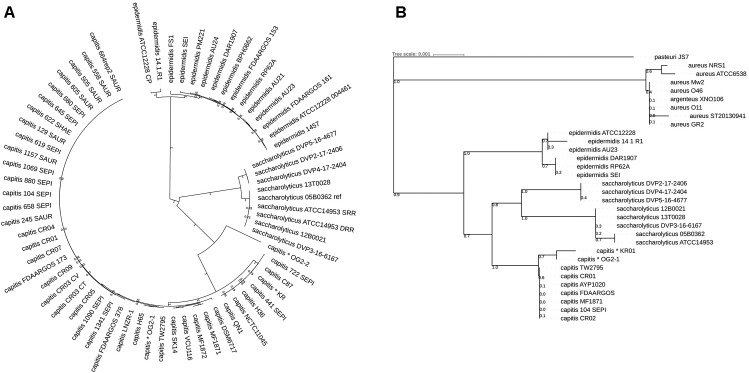
Phylogenetic relationship of staphylococcal species based on core genome-located SNPs and 16S rRNA gene analysis. **(A)** Phylogenic analysis was based on high-quality SNPs in the staphylococcal core genome of all so far sequenced strains of the species *Staphylococcus saccharolyticus*, *Staphylococcus capitis*, and selected strains of *Staphylococcus epidermidis*, using the program Parsnp. All *S. saccharolyticus* strains formed a distinct clade that is clearly distinct from *S. capitis* and *S. epidermidis*. The core genome of the shown strains is 3% (70.5 kb) of the reference genome *S. saccharolyticus* strain 05B0362. The choice of the reference genome had no influence on the outcome of the phylogenetic analysis. The *S. capitis* strains that were previously incorrectly assigned as *S. saccharolyticus* are marked by an asterisk. **(B)** A Blast search with the 16S rRNA gene sequence of *S. saccharolyticus* strain 05B0362 was carried out and the closest matching sequences from other staphylococcal species were extracted and used for phylogenetic reconstruction. Shown are only the strains from which a complete 16S rRNA gene could be retrieved. The evolutionary history was inferred using the Minimum Evolution method. The percentage of replicate trees in which the associated taxa clustered together in the bootstrap test (500 replicates) are shown next to the branches. The tree is drawn to scale, with branch lengths in the same units as those of the evolutionary distances used to infer the phylogenetic tree. Evolutionary analyses were conducted in MEGA7.

Phylogeny was further interrogated by calling single nucleotide polymorphisms (SNPs) in the core genome of the closely related staphylococcal species using Parsnp. Core-genome SNPs were identified and used for phylogenomic reconstruction; this showed that the seven *S. saccharolyticus* strains clustered separately from *S. capitis* and *S. epidermidis* as well as from the previously sequenced strains wrongly assigned to *S. saccharolyticus*, thus supporting the conclusions from the ANI comparison (Figure 1A).

The question remained if the here sequenced strains comprise a new species or if they are “true” *S. saccharolyticus* strains. To interrogate this, we compared our data to the raw sequence data of the type strain of *S. saccharolyticus*, i.e., strain ATCC 14953 (=NCTC 11807); it was sequenced by two independent research teams and the sequence data are available in the sequence read archive (SRA) database and at GenBank (accession number: UHDZ01000000). The assembled two genomes of ATCC 14953/NCTC 11807 were identical. They are also highly similar to the seven here sequenced genomes (ANI of 99%) (Supplementary Table S2 and Figure 1A). We concluded that our strains belong to the species *S. saccharolyticus*. This also meant that so far a genome of this species was never analyzed in any detail before, illustrating the lack of knowledge about *S. saccharolyticus*.

Next, we compared the 16S rRNA sequence of *S. saccharolyticus* with staphylococcal sequences deposited in public databases. The seven *S. saccharolyticus* isolates and the type strain ATCC 14953/NCTC 11807 carry a highly similar 16S rRNA sequence with a total of eight SNPs. 16S rRNA-based phylogeny confirmed the existence of *S. saccharolyticus* as a separate species, and confirmed the misclassification of previously sequenced strains incorrectly assigned to *S. saccharolyticus* (Figure 1B).

### Two Distinct Subclades of *S. saccharolyticus*

A closer inspection of the phylogeny of the *S. saccharolyticus* strains showed that they are grouped into two subclades (Figure 1): one subclade contains five strains (05B0362, 12B0021, 13T0028, DVP3-16-6167, and the type strain ATCC 14953), hereafter called subclade 1; the other subclade contains three isolates (DVP2-17-2406, DVP4-17-2404, and DVP5-16-4677), hereafter called subclade 2. A core genome analysis of all *S. saccharolyticus* strains showed that they share a core genome of 95% with a total number of 33,881 SNPs (data not shown). Strain DVP2-17-2406 is identical to strain DVP4-17-2404; no SNP is present in the core genome; these two strains were isolated in separate tissue biopsies from the same patient.

**FIGURE 2:**
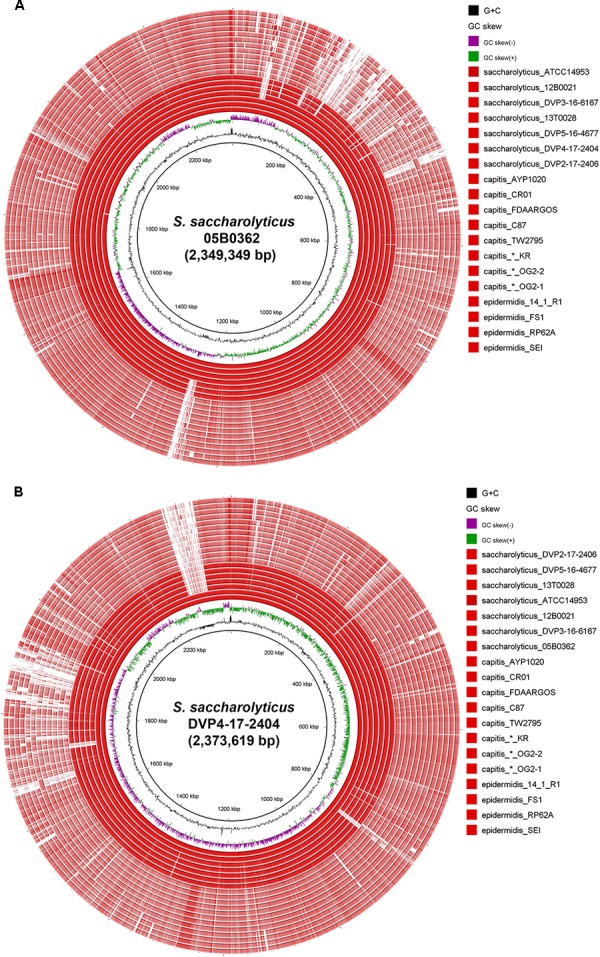
Genome comparison of *S. saccharolyticus* with other staphylococcal species. The two innermost rings represent the G+C-content (black) and the GC–skew (violet/green). **(A)** The reference strain is *S. saccharolyticus* 05B0362 (subclade 1); note the high nucleotide identity, visualized by the dark red color of the seven innermost rings, representing genomes of *S. saccharolyticus*, and the lower identify, visualized by the pale red color of the twelve outer rings [five strains of *S. capitis*, three strains of *S. capitis* that were previously incorrectly assigned as *S. saccharolyticus* (marked by an asterisk) and four strains *S. epidermidis*]. **(B)** the reference strain is *S. saccharolyticus* DVP4-17-2404 (subclade 2). The largest subclade 2-specific region is a 55 kb plasmid (upper left).

Comparative genome analyses were carried out to investigate differences between the two subclades regarding their accessory genomes. Subclade-specific genomic islands were identified. Subclade 1 contained four larger islands (>5 kb) that are lacking in subclade 2 (Figure 2A). These encode: (1) superantigen-encoding pathogenicity island and cadmium resistance (15 kb, not present in subclade 1 strain 13T0028); (2) type I restriction-modification system and *s*taphylococcal cassette *c*hromosome element (9 kb); (3) yersiniabactin synthesis/iron acquisition (6 kb); (4) mobile element-flanked region, possibly encoding biosynthesis of secondary metabolite (20 kb). Subclade 2 strains contain three larger subclade-specific islands (>5 kb) (Figure 2B), encoding: (1) staphylococcal surface anchored protein cluster (10 kb); (2) adhesion proteins and ABC transporter (12 kb); (3) plasmid (55 kb). The plasmid contains the genes for the biosynthesis of a secondary metabolite, generated by hybrid polyketide synthases/non-ribosomal peptide synthetases (PKS–NRPS).

### Genome Differences of *S. saccharolyticus* Compared to *S. capitis* and *S. epidermidis*

Next, we wanted to identify *S. saccharolyticus*-specific genes that are absent from closely related staphylococci in order to determine species-specific traits. All protein sequences of *S. saccharolyticus* were blasted against closely related staphylococci (*S. capitis* and *S. epidermidis*) in a bi-directional manner using the Proteinortho (Supplementary Table S3). Overall, 4,393 coding sequences (CDS) constitute this staphylococcal pan-proteome, and 1,551 CDS are part of the staphylococcal core genome. In average, 2,510 CDS are encoded per individual staphylococcal genome; thus, 62% of the CDS of the average genome is shared among the three species analyzed here.

Regarding *S. saccharolyticus*-specific functions, 221 CDS were identified; these encoded, among others, cell surface (-modifying) components (e.g., adhesins), DNA-modifying factors (e.g., restriction-modification systems), various transporters (e.g., ABC-type transporters) and proteins putatively involved in resistance (Supplementary Table S3). Interestingly, also a gene encoding a hyaluronate lyase was found (see below). We noticed that many *S. saccharolyticus*-specific CDS were smaller fragments of larger, full-length staphylococcal CDS, indicating the presence of premature stop codons; thus, we decided to have a closer look on the presence of pseudogenes in *S. saccharolyticus*.

### Extensive Genome Decay in *S. saccharolyticus*

**FIGURE 3:**
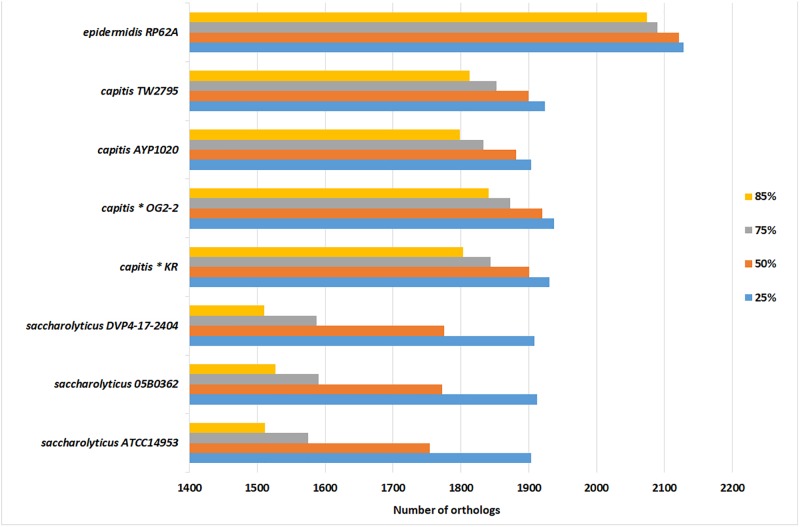
Bi-directional Blast reveals high number of fragmented CDS in *S. saccharolyticus*. *S. epidermidis* ATCC12228 was used as reference genome and the number of orthologs in other closely related staphylococcal species are given, when applying different protein coverage cutoffs (25, 50, 75, and 85%). For instance, *S. saccharolyticus* strain 05B0362 shares 1,912 CDS (with the reference genome) with a minimum protein sequence coverage of 25%, but only 1,527 CDS with a minimum coverage of 85%. In contrast, in *S. capitis* the number of orthologous CDS remained high even at a coverage cutoff of 85%. This indicates gene decay in *S. saccharolyticus*, leading to fragmented CDS. The bi-directional Blast was carried out with the program Proteinortho.

The genome size of *S. saccharolyticus* is in average 2,357 kb, thus smaller that the genome sizes of closely related staphylococcal species. In contrast, surprisingly, more CDS were predicted in *S. saccharolyticus* compared to the other staphylococcal species: the annotation assigned in average 2,711 CDS per *S. saccharolyticus* genome, which is substantially higher than for *S. capitis* (2,374 CDS) and *S. epidermidis* (2,413 CDS) (Table 1 and Supplementary Table S1B). This means that the average length of each CDS must be smaller in *S. saccharolyticus*. To determine this, a bidirectional Blast approach was applied, i.e., the determination of orthologs proteins in different staphylococcal genomes, using four different coverage cutoff values (25, 50, 75, and 85%). The number of orthologs drastically decreased in *S. saccharolyticus* when using a high coverage cutoff (85%). In contrast, in *S. capitis* strains only a moderate decrease in the number of orthologs was detected when using high coverage cutoffs (Figure 3). This indicates extensive gene decay, i.e., the accumulation of pseudogenes in *S. saccharolyticus*. Manual inspection of the annotation data revealed the presence of at least 301 pseudogenes, i.e., genes that were fragmented in *S. saccharolyticus* due to frameshift mutations but were complete in the closely related staphylococcal species *S. capitis* and *S. epidermidis* (Supplementary Table S4). One example is the *ica* gene locus, encoding the biosynthesis of the staphylococcal polysaccharide intercellular adhesin, an important factor for biofilm formation in *S. aureus* as well as in *ica*-positive *S. epidermidis* strains (Cramton et al., 1999; Götz, 2002). Here, each of the genes *icaABCD* is fragmented by one or even multiple frameshift mutations in *S. saccharolyticus* (Supplementary Figure S1). In addition, several metabolic functions are affected by frameshift mutations, such as the amino acid metabolism. According to the KEGG analysis of the pseudogenes, *S. saccharolyticus* is auxotrophic for many amino acids including at least histidine, tryptophan, valine, leucine, isoleucine, methionine, and proline (Supplementary Figure S2). This might explain the slow growth of this species. Alternative growth media and supplements need to be tested in order to determine the nutritional requirements for optimal growth of *S. saccharolyticus*.

### Biochemical Profile of Fastidious *S. saccharolyticus*

To know more about the metabolic capabilities of *S. saccharolyticus*, we tested two commercially available biochemical test systems that are used for the identification of anaerobes. The kit “API^®^ 20A” contains 21 tests, among them 16 tests for carbohydrate utilization. None of the carbohydrates could be metabolized by any *S. saccharolyticus* strain (data not shown). Regarding the other reactions, only gelatin liquefaction was positive, indicating the presence of a protein with gelatinase activity in *S. saccharolyticus*. The kit “RapID^TM^ ANA II” contains 18 tests for enzymatic activities. Saccharolytic enzyme activity was lacking, as well as proteolytic activity (data not shown). The only two positive reactions were phosphatase and urease activity. Interestingly, urease activity was only detected in *S. saccharolyticus* subclade 1 but not in subclade 2 strains. The urease genes could be identified; they encode the alpha, beta and gamma subunits, and highly similar to the urease of the urinary tract pathogen *S. saprophyticus* (Gatermann and Marre, 1989). To explain the lack of urease activity in subclade 2, sequence comparison between subclade 1 and 2 strains was carried out: an insertion mutation in subclade 2 strains was identified that led to a premature stop codon in the *ureB* gene, resulting in a different and shorter C-terminus of UreB (Supplementary Figure S3A). At the same time, this insertion mutation changed the ribosome binding site of *ureA*. It is likely that this mutation is the reason for the lack of urease activity of subclade 2 strains.

### Hyaluronate Lyase Activity of *S. saccharolyticus*

**FIGURE 4:**
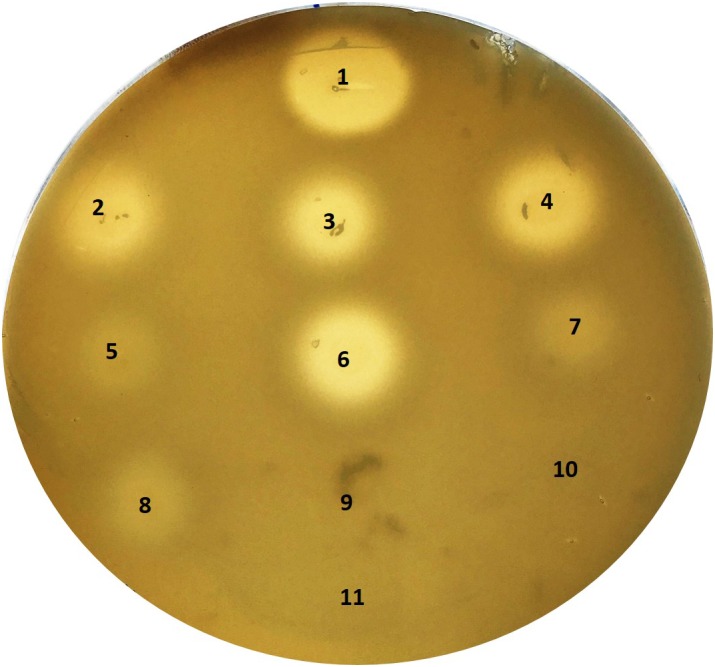
Hyaluronidase activity of *S. saccharolyticus*. Detection of hyaluronate lyase activity with a hyaluronic acid plate assay. Colonies of *S. saccharolyticus* strains were point-inoculated onto hyaluronic acid-containing plates and incubated for 48 h under anaerobic conditions. Plates were flushed with 2N acetic acid for 15 min for the detection of hyaluronic acid degradation. Numbers (in brackets the respective *S. saccharolyticus* subclade): 1, positive control (hyaluronidase from *Streptococcus pyogenes*); 2, 05B0362 (1); 3, 12B0021 (1); 4, 13T0028 (1); 5, DVP2-17-2406 (2); 6, DVP3-16-6167 (1); 7, DVP4-17-2404 (2); 8, DVP5-16-4677 (2); 9, *S. epidermidis* FSI; 10, *S. epidermidis* 14.1.R1; 11, BHCY medium (negative control). The pictures are representative of three independent experiments.

Among the genes present in *S. saccharolyticus* but absent in *S. capitis* and *S. epidermidis* a gene for hyaluronate lyase/hyaluronidase (*hysA*) was found (Supplementary Table S3). The HysA protein, 799 amino acids large, harbored an N-terminal signal peptide for protein export. Highest similarity exists to homologs in *Staphylococcus agnetis* and *Staphylococcus hyicus* (each 65% identity on protein level), and *S. aureus* (57%). The hyaluronidase cleaves the hyaluronic acid polymer at the β-1,4 glycosidic bond; it was reported as a virulence factor for *S. aureus*, important in the early stages of subcutaneous infections (Makris et al., 2004). We therefore checked for hyaluronidase activity of the *S. saccharolyticus* strains, applying an agar plate assay. Results showed that all strains of *S. saccharolyticus* exhibited hyaluronidase activity, in contrast to *S. epidermidis* (Figure 4). However, subclade 1 strains had a stronger activity compared to subclade 2 strains that showed only weak but detectable activity. To understand this difference, we compared the *hysA* gene locus in the two subclades, and identified a 13-bp deletion in the *hysA* promoter region of subclade 2 strains, that fell within the predicted -35 region of the promoter (Supplementary Figure S3B). Moreover, a base substitution in the -10 region of the promoter was detected in subclade 2 strains. It is likely that these differences affect the transcription efficiency of *hysA*.

**FIGURE 5:**
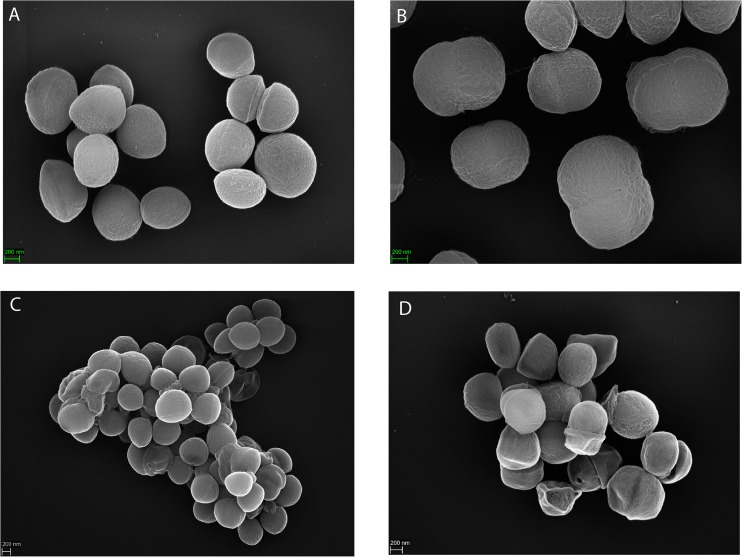
Scanning electron microscopy of *S. saccharolyticus*. The upper **(A,B)** and lower **(C,D)** panels show representative images of *S. saccharolyticus* 13T0028 (subclade 1) and *S. saccharolyticus* DVP4-17-2404 (subclade 2), respectively. Images indicate morphological differences between these two strains.

The subclade-specific hyaluronidase and urease activities suggest further substantial differences between subclades 1 and 2 strains. Indeed, also electron microscopy indicated differences in the morphology and cell arrangement (Figure 5). Cells of strain 13T0028 (subclade 1) were arranged more individually, whereas cells of strain DVP4-17-2404 (subclade 2) were arranged in grapelike clusters and showed more signs of cell surface damage.

### Secretome of *S. saccharolyticus*

Given the above-mentioned results, including the accumulation of pseudogenes, the peculiar biochemical profile and the urease and hyaluronidase activities, we decided to determine the secreted proteins of this species in order to get a better picture of the possible degradative and metabolic capabilities and the host-interacting potential. Two strains, one of each subclade, were grown in complex medium under anaerobic conditions. Cultures were harvested at two time points (4 and 7 days of growth). Supernatant were harvested and secreted proteins determined by mass spectrometry. In average, 98 and 141 proteins were identified in the supernatants at the early (4 days) and late (7 days) time point, respectively. No major qualitative differences were observed between the secretomes of the two strains. Also, no major changes were detected between the two time points: largely the same proteins with highest identification scores were detected at days 4 and 7 (Supplementary Table S5).

Stress response proteins, including heat-shock proteins such as the chaperones GroEL, GroES and DnaK and oxidative stress-related proteins including alkyl hydroperoxide reductase protein C, thioredoxin and superoxide dismutase were detected with highest identification scores, indicative of their abundance in the culture supernatants (Table 2 and Supplementary Table S5). Another class of abundantly detected proteins were enzymes of the glycolysis, including glyceraldehyde 3-phosphate dehydrogenase (GAPDH) and enolase, as well as enzymes of the arginine deiminase system involved in acid tolerance, including ornithine carbamoyltransferase and arginine deiminase. Most of the above-mentioned proteins are cytosolic proteins, indicating that partial cell lysis takes place even in the early growth phase. However, enzymes such as GAPDH, enolase and GroEL are reported to be surface-exposed or secreted proteins also in other Gram-positive species and are considered moonlighting proteins (Henderson and Martin, 2013; Ebner and Götz, 2019). Excretion of cytoplasmic proteins has also been found in *S. aureus* (Ebner et al., 2015; Ebner et al., 2017).

**Table 2:** Selected proteins of *S. saccharolyticus* (strain 13T0028) identified in the culture supernatant.

Locus tag	Annotation	Mass KDA	Score^∗^	Coverage %	Unique peptides	Score^∗^ in DVP4-17-2404
13T0028_PEG.2671	Heat shock protein 60 chaperone GroEL	57.5	1617	33.2	17	2716
13T0028_PEG.1579	Enolase	47.2	1479	45.2	17	1496
13T0028_PEG.1550	Putative extracellular amidase	29.4	1308	39.8	7	583
13T0028_PEG.415	Glycerol dehydrogenase	40.1	1053	35.9	10	1448
13T0028_PEG.411	Ornithine carbamoyltransferase	37.8	915	33	11	776
13T0028_PEG.483	Triacylglycerol lipase	79.4	907	21.2	14	589
13T0028_PEG.1013	Thioredoxin	11.5	729	75	8	257
13T0028_PEG.1809	DNA-binding protein HBsu	9.6	692	63.3	5	1489
13T0028_PEG.1920	Chaperone protein DnaK	65.8	684	18.3	12	799
13T0028_PEG.690	Immunodominant staphylococcal antigen A	24.0	667	8.7	3	392
13T0028_PEG.2672	Heat shock protein 60 co-chaperone GroES	10.2	619	58.5	5	619
13T0028_PEG.140	Alkyl hydroperoxide reductase protein C	21.1	594	39.2	6	706
13T0028_PEG.1575	NAD-dependent GAPDH	36.3	497	27.4	9	879
13T0028_PEG.1766	Bifunctional autolysin Atl/*N*-acetylmuramoyl-L-alanine amidase/endo-beta-*N*-acetylglucosaminidase	148.5	456	7.6	11	–
13T0028_PEG.546	Arginine deiminase	47.254	248	18.2	7	681
13T0028_PEG.1893	Manganese superoxide dismutase	22.658	222	24.7	4	256
13T0028_PEG.1381	Staphylococcal accessory regulator A (SarA)	14.835	188	23.4	3	212
13T0028_PEG.2613	Immunodominant antigen B	20.754	162	10.9	2	173
13T0028_PEG.433	Triacylglycerol lipase	81.495	147	5.1	3	93
13T0028_PEG.1825	Staphylococcal respiratory response protein SrrA	28.073	73	10.8	2	–
13T0028_PEG.326	Hyaluronate lyase	92.1	53	1.9	2	46


*^∗^MASCOT score*.

Some macromolecule-degrading enzymes were found, including two triacylglycerol lipases, an amidase (possible autolysin) and the above-mentioned hyaluronate lyase. These degradative enzymes possess characteristic N-terminal signal peptides for protein export. Among possible host immune system-interacting factors both, immunodominant staphylococcal surface antigens A (IsaA) and B (IsaB) were found as secreted proteins. Interestingly, regulators that are part of the *staphylococcal* quorum-sensing system were also found, i.e., SarA and SrrA. Both proteins are important regulators of staphylococcal virulence factors, activated in response to, e.g., low oxygen levels (Cheung et al., 1992; Pragman et al., 2004).

## Discussion

A cohort of bacterial strains, identified as *S. saccharolyticus* by MALDI-TOF MS, isolated from PJIs and blood cultures of patients at the Örebro University Hospital, Sweden, were analyzed in the present study with the initial aim to gain more insight into the genome and microbiology of *S. saccharolyticus*.

Our data revealed a taxonomic problem within CoNS. Three strains, isolated from kefir, have previously been sequenced that were classified as *S. saccharolyticus*. The genomic data clearly showed that at least two of the three strains should be reclassified as *S. capitis*. The main reason for this misidentification was the lack of a reference genome sequence of *S. saccharolyticus.* Here, we closed this knowledge gap by sequencing and analyzing several genomes of *S. saccharolyticus*.

Previous observations pointed to difficulties to distinguish *S. saccharolyticus* from *S. capitis*; e.g., it was noted that strains of *S. saccharolyticus* and *S. capitis* are indistinguishable as judged from biochemical tests (Evans et al., 1978; Steinbrueckner et al., 2001). Our data now clearly showed that *S. saccharolyticus* is a distinguishable, individual species, based on the following observations:

(1) The genome-wide ANI of *S. saccharolyticus* to the closest relatives, i.e., *S. capitis* and *S. epidermidis*, was only approximately 80%. According to recommendations to define species based on DNA similarity, an ANI below 95% is regarded as a good indication of species separation (Richter and Rosselló-Móra, 2009).
(2) Genome size, GC-content and 16S rRNA sequence of *S. saccharolyticus* differ substantially from closely related CoNS.
(3) *S. saccharolyticus* has some properties that are unusual for other CoNS: strains of *S. saccharolyticus*, in particular those of subclade 1, exhibit urease and hyaluronidase activities.
(4) The genome of *S. saccharolyticus* is peculiar: it contains about 300 pseudogenes, indicative of ongoing reductive evolution. This has so far not been described for other staphylococci, with the exception of *Staphylococcus carnosus*, whose genome contains 55 truncated genes (Rosenstein et al., 2009).

We also noticed the existence of two subclades among the *S. saccharolyticus* strains. These subclades differed not only in the core genome (many SNP differences) but also in their flexible genomes, which indicates that they represent two individual subspecies of *S. saccharolyticus* that exhibit also biochemical and, possibly, morphological differences.

Some interesting features of *S. saccharolyticus* have been identified in our study, including urease and hyaluronidase activities. For *S. saccharolyticus* subclade 1 strains urease activity could be detected. The multi-subunit enzyme complex catalyzes the hydrolysis of urea, leading to ammonia and carbon dioxide formation; thus, it is important for pH increase. The corresponding genes are found in all *S. saccharolyticus* genomes; *ureABC* encode the different subunits (α, β and γ) and *ureDEFG* encode the accessory proteins (Burne and Chen, 2000). Urease has been identified as a virulence factor for the Gram-positive urinary tract pathogen *S. saprophyticus* and other bacteria such as *Helicobacter pylori* (Burne and Chen, 2000; Loes et al., 2014).

Regarding pH regulation, we noticed another system present in all *S. saccharolyticus* strains: the arginine deiminase (ADI) pathway. This pathway catalyzes the conversion of arginine to ornithine via citrulline, employing the enzymes arginine deiminase, ornithine carbamoyltransferase and carbamate kinase, thereby generating NH_3_ and ATP. Both, arginine deiminase and ornithine carbamoyltransferase are abundantly detected in the secreted fraction. This system is used to counteract the acidification of the culture medium in the course of anaerobic fermentation, thus facilitating survival in acidic conditions (Gruening et al., 2006). The ADI pathway was also reported to function as an additional energy-conserving pathway during anaerobic growth (Valgepea et al., 2017).

All *S. saccharolyticus* strains exhibited hyaluronidase activity, with subclade 1 strains exhibiting a superior activity compared to subclade 2 strains. Hyaluronidases degrade hyaluronic acid, a major polysaccharide of the extracellular matrix of tissues; the enzyme is a virulence factor in a number of Gram-positive bacteria (Hynes and Walton, 2000; Makris et al., 2004; Ibberson et al., 2014). The enzyme, encoded by the *hysA* gene, is produced by most *S. aureus* strains and it was suggested that only *S. aureus* possesses the enzyme among human-associated staphylococci (Hart et al., 2009). This now has to be rectified. The closest homologs of HysA of *S. saccharolyticus* can be found in *S. agnetis* and *S. hyicus*; these are both animal pathogens. *S. agnetis* is associated with lameness in broiler chickens, and is isolated from cases of endocarditis and septicemia in those animals; *S. hyicus* causes skin diseases, such as exudative dermatitis in piglets (Hazarika et al., 1991; Foster, 2012).

A surprising feature of all *S. saccharolyticus* genomes was the high number of pseudogenes, indicative of ongoing genome decay. The inactivation of genes is often the result of a lifestyle switch, including the adaption to a new niche. This has been seen in other bacteria that relatively recently changed their life style and/or host association, e.g., from a free-living to a strictly host-associated lifestyle (Pallen and Wren, 2007). The most prominent example is the very slow-growing, leprosy-causing bacterium *Mycobacterium leprae*, but genome decay is seen also in other pathogens such as *Yersinia pestis* and *Salmonella enterica* serovar Typhi, as well as in several host-associated bacteria, particularly endosymbionts and/or intracellular bacteria (Cole et al., 2001; Parkhill et al., 2001a,b; Pallen and Wren, 2007).

It is apparent from the secretome data that *S. saccharolyticus* did not ‘feel’ comfortable as a free-living bacterium with the provided growth conditions (medium, temperature, and cultivation conditions). Many secreted proteins are actually cytosolic stress response proteins that have functions to counteract acidic, temperature and oxidative stress. Thus, it is likely that *S. saccharolyticus* has other preferred growth conditions and/or is depended on the host for optimal proliferation. This stress response could also be intrinsic due to extensive genome decay that led to the inactivation of staphylococcal core functions. For example, several amino acid biosynthesis genes are apparently not functional due to premature stop codons; thus, it can be predicted that the organism is auxotrophic for several amino acids, including histidine, tryptophan, valine, leucine, isoleucine, methionine and proline. This is in analogy to *S. carnosus* (Rosenstein et al., 2009). In addition, it could be hypothesized that the dependence of *S. saccharolyticus* on anaerobic growth conditions is a result of loss of function mutations. However, no obvious frameshift mutations or premature stop codons were found in the genes of the respiratory chain or oxygen detoxification systems, such as catalase and superoxide dismutase (SOD). The latter gene had a different 3′end compared to SODs of other staphylococci, including *S. epidermidis, S. capitis*, and *S. aureus* (data not shown). It should be mentioned that the dependence of *S. saccharolyticus* on anaerobic conditions is not strict: whereas primary isolates grow only under anaerobic conditions, slow growth upon sub-cultivation can be observed in an aerobic atmosphere with 10% CO_2_ (Evans et al., 1978).

In the future, host-interactions of *S. saccharolyticus* need to be investigated in order to determine its pathogenicity. Some features, including urease and hyaluronidase activities suggest an invasive capability of *S. saccharolyticus*. In addition, also immune system-modulating proteins such as the immunodominant staphylococcal surface antigens A (IsaA) and B (IsaB) are found among the secreted proteins. IsaA has been described as lytic transglycosylase in *S. aureus* and IsaB is a putative nucleic acid-binding virulence factor of *S. aureus* (Stapleton et al., 2007; Mackey-Lawrence et al., 2009). A recent study showed that it helps evade the host response; more specifically, IsaB can inhibit the autophagic processing, thus allowing *S. aureus* to evade host degradation (Liu et al., 2015).

However, no additional genes encoding well-characterized virulence factors of *S. aureus* are found in the genome of *S. saccharolyticus*, with the exception of six low-molecular-weight toxins of the phenol-soluble modulin (PSM) family (five β-type and one δ-type PSMs). PSMs have diverse functions in growth, colonization/biofilm, inflammation and cytotoxicity and carry out roles in commensal as well as pathogenic staphylococcal lifestyles (Cheung et al., 2014). PSMs are regulated by the global regulatory quorum sensing system Agr (accessory gene regulator) in a cell density-dependent manner. The genome of *S. saccharolyticus* contains an apparently functional *agr* locus (*agrBDCA*); the autoinducing peptide (AIP, encoded by *agrD*) is a type I AIP, as judged from the comparison with AIPs of *S. epidermidis* (Olson et al., 2014).

Other quorum-sensing systems were found in *S. saccharolyticus*, such as the SrrAB system; interestingly, its regulator SrrA was detected in the secretome. The SrrAB system coordinates a stress response under hypoxic conditions; it was found to be essential for survival of *S. aureus* during invasive infections (Pragman et al., 2004; Wilde et al., 2015). Furthermore, virulence, biofilm formation and cytotoxicity of *S. aureus* and *S. epidermidis* were found to be increased under hypoxic conditions (Cramton et al., 2001; Wilde et al., 2015; Balasubramanian et al., 2017). It could be hypothesized that the dependence on anaerobic conditions of *S. saccharolyticus* implies inherently elevated virulence (in a quorum-sensing-dependent manner), thus influencing the ability to invade human tissue and colonize medical devices.

Questions regarding the preferred ecological niche of *S. saccharolyticus* and its frequencies were not addressed in this study. In a study of 1978, *S. saccharolyticus* was identified on the skin of the forehead and the antecubital fossa of the arm of ca. 20% of the human subjects studied; identification was based on biochemical tests and culture characteristics (Evans et al., 1978). It is not possible to determine retrospectively, if the identified isolates all truly belonged to *S. saccharolyticus*. Sequences searches using the 16S rRNA sequence of *S. saccharolyticus* identified hundreds of 100% identical matches in public databases, in particular to sequences obtained in human (skin) metagenome projects; the matching 16S rRNA sequences belonged to organisms labeled as “uncultured bacterium” or “*Staphylococcus* sp.” (data not shown). This indicates that *S. saccharolyticus* is common in humans, with human skin as preferred habitat.

## Conclusion

We have analyzed for the first time the (pan-)genome of *S. saccharolyticus* and found several peculiarities that are not found in other human-associated CoNS. The species has apparently undergone a relatively recent lifestyle change, illustrated by ongoing genome decay. Several indications suggest that *S. saccharolyticus* has host tissue-invasive potential and is associated with prosthetic joint infections.

## Data Availability

The datasets generated and analyzed for this study can be found at GenBank (https://www.ncbi.nlm.nih.gov/genbank/) (GenBank accession numbers are: strain 05B0362, QHKH00000000; strain 12B0021, QHKG00000000; strain 13T0028, QHKF00000000; strain DVP3-16-6167, QHKE00000000; strain DVP2-17-2406, QHKD00000000; strain DVP4-17-2404, QHKC00000000; and strain DVP5-16-4677, QHKB00000000).

## Author Contributions

BS and HB conceived and designed the study. AP and EB performed genome sequencing and primary sequence data analyses. CS and JE performed proteome analyses. MA-Z and VB performed scanning electron microscopy experiments. BS provided strains and performed MALDI-TOF MS. HB and AJ analyzed genomic data. HB performed biochemical experiments and wrote the manuscript.

## Conflict of Interest Statement

The authors declare that the research was conducted in the absence of any commercial or financial relationships that could be construed as a potential conflict of interest.

## Footnotes

1 https://itol.embl.de/
