# Supplementary material for: Staphylococcus saccharolyticus Isolated From Blood Cultures and Prosthetic Joint Infections Exhibits Excessive Genome Decay
Source: Front Microbiol. 2019 Mar 12;10:478. doi: 10.3389/fmicb.2019.00478 (PMC6423177; doi:10.3389/fmicb.2019.00478)
Supplement: Supplementary file 6 [file Data_Sheet_1.PDF]

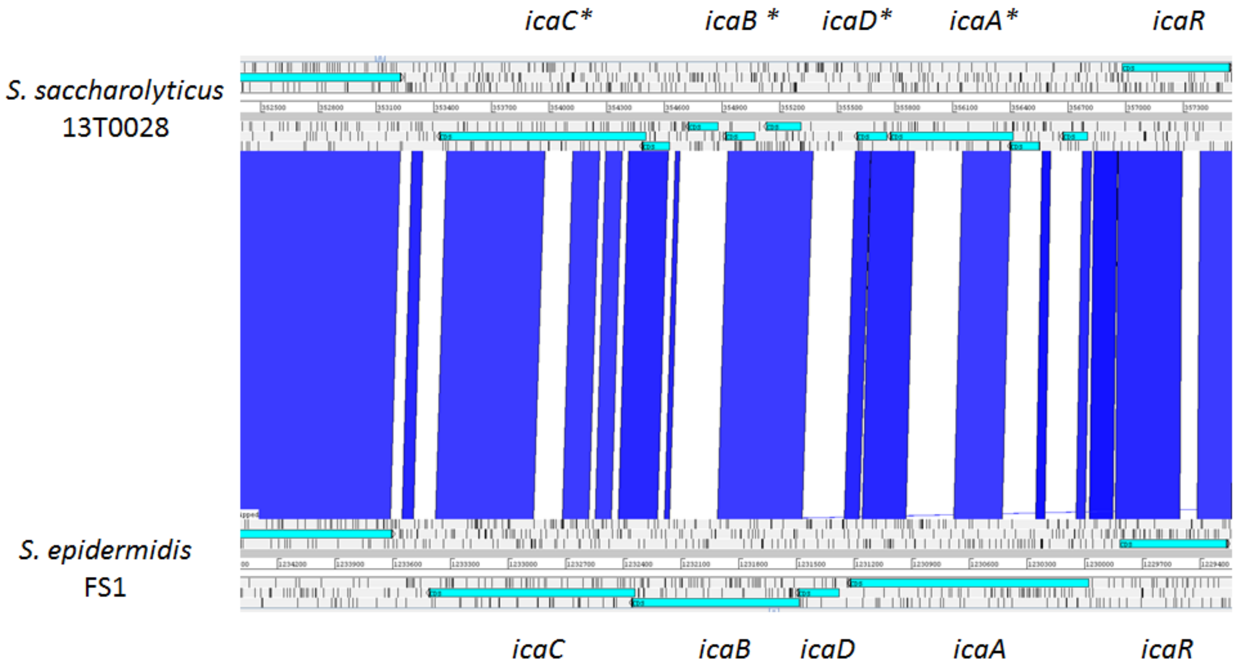

Figure S1: Fragmentation of the *ica* gene locus in *S. saccharolyticus*
Frameshift mutations were detected in all four *ica* genes (*icaABCD*) in *S. saccharolyticus* strain 13T0028 as well as in all other *S. saccharolyticus* strains.

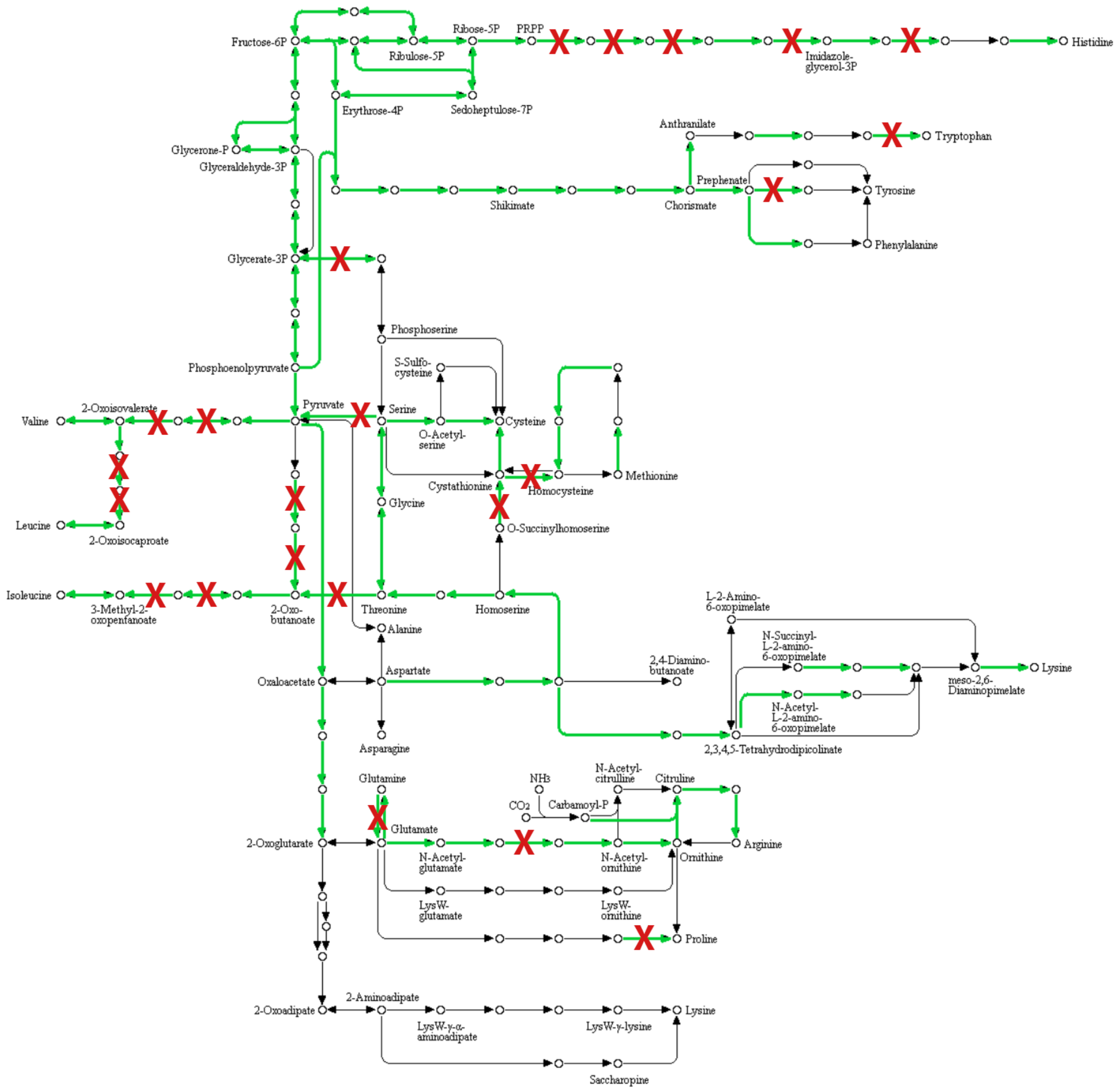

Figure S2: Limited capability of *S. saccharolyticus* to biosynthesize amino acids due to genome decay
Red crosses on green arrows indicate those reactions that are inactivated due to frame-shift or nonsense mutations in the corresponding genes. Green arrows without red crosses indicate existing, functional reactions. Black arrows represent those reactions that were not found to be encoded in the genome.

A

Subclade 1 of *S. saccharolyticus*:

```
UreB
G  E  E  N  A  N  K  K  G  G  N  Q  A  #
GGCGAAGAAAACGCAAATAAAAAAGGTGGTAATCAAGCATGAGTTTCAAAATGACACAATCTCAATAC
                                      M  S  F  K  M  T  Q  S  Q  Y
                                                                UreA
```

Subclade 2 of *S. saccharolyticus*:

```
UreB
G  E  K  N  A  N  K  K  G  R  #
GGCGAAAAAAACGCAAATAAAAAAGGTAGGTAATCAAGCATGAGTTTCAAAATGACACAATCTCAATAC
                                       M  S  F  K  M  T  Q  S  Q  Y
                                                                 UreA
```

B

Subclade 1 of *S. saccharolyticus*:

```
AGCAAGATATTTCAACAAGTTAATTGAACAAGATCCTCATCATATTAAATTTAGAAAGATAAATCATCAATGTTA
AGGAGATGCCTCAATG
```

Subclade 2 of *S. saccharolyticus*:

```
AGCAAGATATTTC-------------AACAAGATCCTCATCATATTAAGTTTAGAAAGATAAATCCTCAATGTTA
AGGAGATGCCTCAATG
```

Figure S3: Sequence differences between *S. saccharolyticus* subclade 1 and 2 affect the genes coding for urease and hyaluronate lyase

A. An insertion mutation is detected in the 3'-end of the urease beta subunit-encoding *ureB* of subclade 2 strains. This has two consequences: 1. The mutation leads to a premature stop codon (in purple) in *ureB* that results in a changed and shorter C-terminus of UreB. 2. The insertion mutation is within the Shine-Dalgarno sequence (in yellow) of *ureA* and is likely to change the ribosome-binding properties.

B. A deletion of 13 bases is detected in the upstream region of the hyaluronate lyase gene (*hyl*) in subclade 2 strains. This affects the -35 region of the predicted promoter (in green). In addition, a mutation in the predicted -10 region of the promoter is detected (in purple). Both changes are likely to decrease *hyl* transcription in subclade 2 strains.
